# Imaging and histological features of tumor biopsy sample predict aggressive intrasegmental recurrence of hepatocellular carcinoma after radiofrequency ablation

**DOI:** 10.1038/s41598-022-23315-5

**Published:** 2022-11-04

**Authors:** Elia Gigante, Yohann Haddad, Jean-Charles Nault, Olivier Sutter, Einas Abou Ali, Baptiste Bonnet, Gisèle N’Kontchou, Veronique Grando, Nathalie Ganne-Carrié, Pierre Nahon, Lorraine Blaise, Julien Calderaro, Nathalie Barget, Olivier Seror, Marianne Ziol

**Affiliations:** 1 Service d’hépatologie, Hôpital Avicenne, Hôpitaux Universitaires Paris-Seine-Saint-Denis, Assistance Publique Hôpitaux de Paris, Bobigny, France; 2 Université Sorbonne Paris Nord, 93000 Bobigny, France; 3 Unité de Radiologie Interventionnelle, Hôpital Avicenne, Hôpitaux Universitaires Paris-Seine-Saint-Denis, Assistance-Publique Hôpitaux de Paris, Bobigny, France; 4 Centre de Recherche des Cordeliers, Sorbonne Université, Inserm, USPC, Université de Paris, Functional Genomics of Solid Tumors, 75006 Paris, France; 5 Département de Pathologie, Hôpital Henri Mondor, Assistance Publique Hopitaux de Paris, Université Paris Est Créteil, Inserm U955, Paris, France; 6 Centre de ressources biologiques (BB-0033-00027) Hôpitaux Universitaires Paris-Seine-Saint-Denis, Assistance-Publique Hôpitaux de Paris, Bobigny, France; 7 Service d’anatomie Pathologique, Hôpital Avicenne, Hôpitaux Universitaires Paris-Seine-Saint-Denis, Assistance-Publique Hôpitaux de Paris, 125 rue de Stalingrad, 93000 Bobigny, France

**Keywords:** Cancer imaging, Cancer therapy, Liver cancer, Hepatocellular carcinoma, Tumour biomarkers

## Abstract

Aggressive intrasegmental recurrence (AIR) is a form of local recurrence associated with a dismal prognosis and defined by multiple nodules or by an infiltrative mass with a tumor thrombus, occurring in the treated segment, after radiofrequency ablation (RFA) for hepatocellular carcinoma (HCC). We aimed to identify radiological and/or histological characteristics of tumor biopsy predictive of AIR. We retrospectively analyzed patients treated by No-Touch multi-bipolar RFA (mbpRFA) for a first HCC with a systematic per-procedural tumor biopsy positive for diagnosis of HCC. The first recurrence was classified as non-aggressive local recurrence, AIR or intrahepatic distant recurrence. 212 patients were included (168 men; mean age 67.1 years; mean tumor size 28.6 mm, 181 cirrhosis). AIR occurred in 21/212 patients (10%) and was associated with a higher risk of death (57% in patients with AIR vs 30% without AIR, p = 0.0001). Non-smooth tumor margins, observed in 21% of the patients and macro-trabecular massive histological subtype, observed in 12% of the patients were independently related to a higher risk of AIR (HR: 3.7[1.57;9.06], p = 0.002 and HR:3.8[2.47;10], p = 0.005 respectively). Non smooth margins at imaging and macro-trabecular massive histological subtype are associated with AIR and could be considered as aggressive features useful to stratify therapeutic strategy.

## Introduction

Radiofrequency ablation (RFA) is one of the main curative treatment for early-stage hepatocellular carcinoma (HCC) as defined by the Barcelona Clinic Liver Cancer (BCLC) strategy^1,2^. According to previous studies comparing RFA and surgical resection, RFA provided around 70% of overall survival at 3 years, that was similar to those obtained with surgical hepatic resection in patients with early-stage HCC^3–5^. Up to fifteen to 25% of local tumor recurrences have been reported in RFA series, but most of these recurrences are spatially limited to the margin of the ablation zone and can be successfully treated by an additional procedure with a limited impact on overall survival^6–8^. We previously developed a new technique of centripetal percutaneous ablation called the “No Touch multi-bipolar RFA” (No Touch mbpRFA), that was shown to decrease local recurrence compared to monopolar RFA, able to successfully treat more than 3 cm large HCC^9–11^. No Touch mbpRFA is able to treat a wider spectrum of HCC according to their size, shape and location, compared to usual centrifugal energy radiating techniques^12^.

An aggressive form of local recurrence, called aggressive intrasegmental recurrence (AIR), has been described after monopolar RFA performed for early HCC^13^. AIR is defined by the simultaneous development, in the treated segment, of multiple recurrent nodules (at least 3) uniform in size, or by a diffuse infiltrative mass accompanied by a tumor thrombus in the adjacent portal vein^13^. AIR is most often not eligible for re-treatment in a curative attempt and, consequently, strongly impacts the survival of patient^13^. It has been hypothesized that AIR could be related to the vascular spread of an incompletely ablated tumor close to large vessels and/or to intrinsic tumor or stromal features of aggressiveness. Microvascular invasion is a recognized feature of aggressiveness, but cannot be evaluated on biopsy samples. Therefore, alternative markers of aggressiveness based on imaging or histological need to be identified to predict HCC recurrence after ablation and more precisely, to predict the pattern of tumor recurrence that critically impact survival^14,15^. The high local efficacy of No Touch multi-bipolar RFA for the treatment of HCC up to 5 cm might limit the influence of technical failures and therefore allow focusing on biological tumor behavior in occurrence of AIR. To explore this hypothesis, taking the opportunity that since 2007, our standard protocol for percutaneous ablation of HCC includes a per procedural biopsy, we aimed to identify radiological and/or histological characteristics predictive of AIR in patients treated by No Touch mbpRFA for histologically proven HCC.

## Materials and methods

### Patients

**Figure 1:**
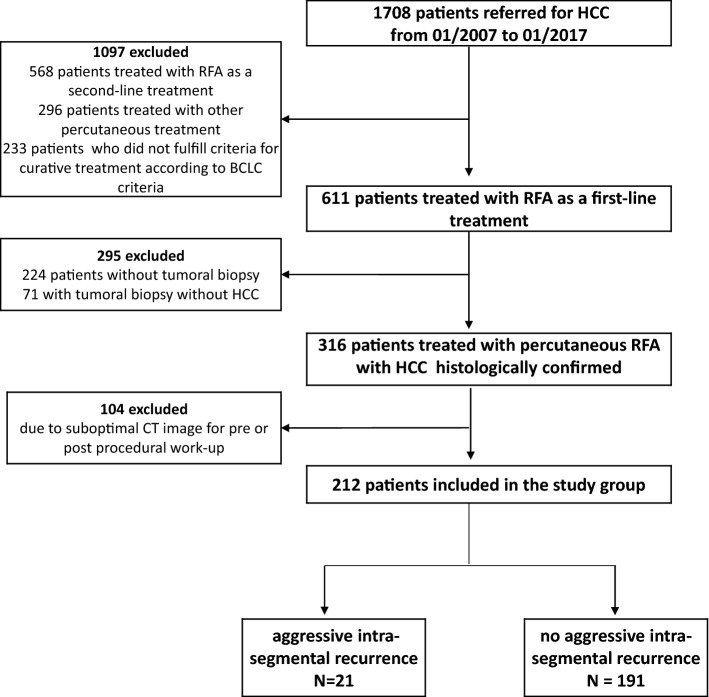
Flow-chart of the study.

We retrospectively selected patients referred for HCC to the weekly multidisciplinary liver tumor board of our University Hospital from January 2007 to June 2017. We included in this study all the patients treated for a first HCC by percutaneous No Touch mbpRFA, with an available histological per procedural biopsy sample confirming the HCC and with an optimal conventional triphasic imaging available (Fig. 1). All patients included were discussed in the multidisciplinary board and were not eligible for surgery. The following variables were recorded at the time of RFA: sex, age, etiology of the chronic liver disease, histological diagnosis of cirrhosis, standard clinical, radiological, biological, histological data and follow-up were also recorded.

### Multibipolar RFA procedures

All No Touch mbpRFA procedures were performed percutaneously by experienced operators. Ultrasonography (US) alone or US fusion imaging with CT or MR was used for imaging guidance. As previously described, multi-bipolar RFA were performed using a multi-channel 250-W (maximal output power) 470-kHz radiofrequency generator (CelonLabPower; OlympusCelon)^9^.

### Imaging and follow-up

**Figure 2:**
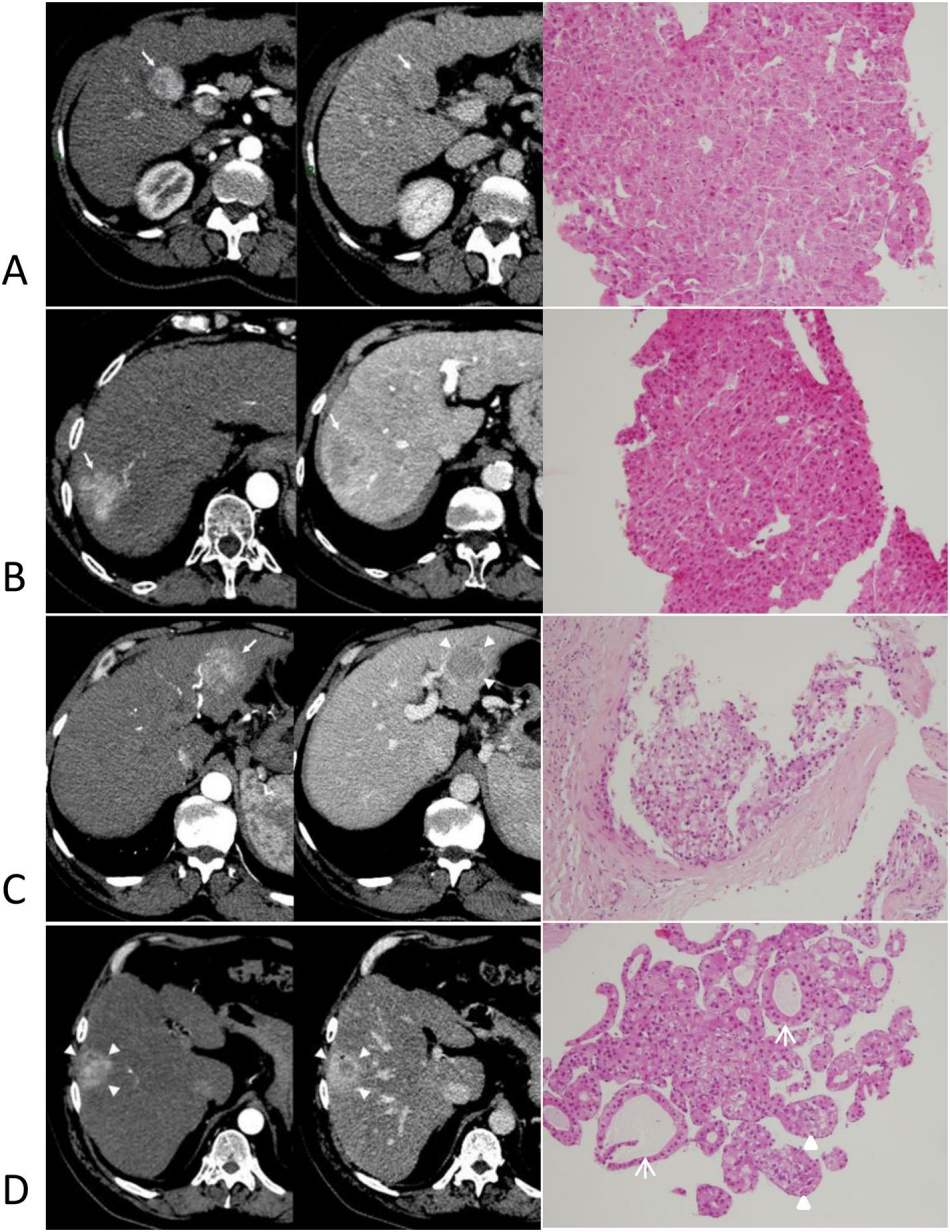
Imaging and histological features of hepatocellular carcinomas at the time of diagnosis. Axial CT scans showing hepatocellular carcinomas (arrows) with typical hypervascular pattern at arterial phase (left parts) and washout at portal phase (right parts), with corresponding histology of the tumor biopsy (hematein eosin and saffron staining × 100 magnification). Line (**A**) shows a unique nodule with smooth margin. The liver biopsy showed a well differentiated, Edmondson grade 2 HCC with microtrabecular architecture. Line (**B**) shows non smooth margins. Histology showed a moderately differentiated, Edmondson grade 3 HCC with microtrabecular architecture. Line (**C**) shows smooth margins and a capsule on the portal phase (right part, arrowheads) and a continuous linear-enhancing rim around the tumor. The liver tumor biopsy showed a scirrhous HCC with clear cells. Line (**D**) shows a nodule with smooth margins and an abnormal peritumoral arterial enhancement that is still slightly visible on the portal phase (right part, arrowheads). Liver biopsy shows an HCC with pseudoglands (arrows) and more than 6 cell large clusters of tumor cells surrounded by sinusoïdal cells and separated by empty spaces, that defined the macrotrabecular-massive subtype (arrowheads).

Imaging was performed using either computed tomography (CT) (Brilliance 64; Philips) or magnetic resonance (MR) imaging (1.5-T Intera; Philips). The imaging protocol included unenhanced, arterial, and equilibrium phases liver acquisitions after the injection of intravenous contrast medium (iodinated or gadolinated) with an automatic injector triggered at the arterial phase by bolus detection in the aorta. Equilibrium phases were acquired from 2 to 3 min after the beginning of contrast medium injection. All pretherapeutic cross-sectional CT or MR imaging studies with contrast medium intravenous injection were reviewed and choosing the most relevant phases, the following criteria (Fig. 2) were recorded: (a) the number of tumors, (b) the maximal size of the largest tumor, (c) the pattern of tumor enhancement which was considered as typical if both arterial phase hyperenhancement on arterial phase and wash-out on portal or equilibrium phases were present, and atypical if any of these characteristics lacked, (d) the tumor margin which were categorized as smooth or non-smooth according the sharpness of the demarcation of the tumor from the surrounding liver parenchyma; multinodular confluent nodules or infiltrative tumor were regarded systematically as non-smooth margin tumors, (e) the presence of a tumor capsule seen on the delayed phase as a continuous linear-enhancing rim around the tumor, (f) presence of abnormal peritumoral arterial enhancement which was defined by the presence of any peripheral enhancement on arterial-phase images (still visible or disappearing on equilibrium-phase images). In addition, tumors were regarded as perivascular when the tumor presented any contact with a first- or second-degree branch of a portal or hepatic vein larger than 3 mm in diameter.

**Figure 3:**
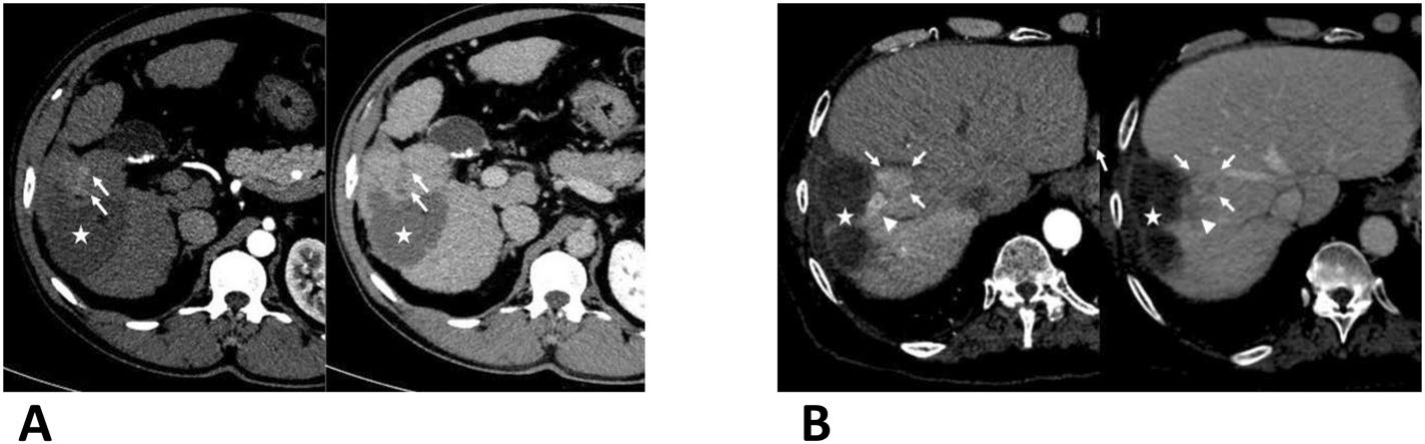
Imaging features of aggressive intrasegmental recurrences. Axial CT scans showing the two patterns of aggressive intrasegmental recurrences (arrows) adjacent to ablation zone (stars), at arterial (left parts) and equilibrium phases (right parts), (**A**) simultaneous development of multiple nodular (at least three, the 3rd nodule is outside the plan of this slice) or (**B**) infiltrative mass in the treated segment associated with tumoral invasion of segmental and/or sectorial portal branches (arrowheads).

These criteria were assessed by 2 radiologists (YH and BB) and interobserver agreement was reported. In case of disagreement, cases were reviewed with a senior radiologist (OS) to reach an agreement. Patients were followed up according to the same schedule with serum biological tests and at least standard triphasic cross-sectional imaging examinations with either computed tomography (CT) or magnetic resonance (MR) imaging one month after treatment. When the ablation, according to standard terminology criteria^16^, was considered as complete (up to three additional RFA procedures if necessary) follow-up was continued every 3 months for 2 years, and every 6 months thereafter. The treatment was considered successful when no local tumor progression was detected at first control imaging. The type of recurrence was classified on the first post ablation CT or MRI into 3 categories as follows: AIR, non-aggressive local recurrence, and intrahepatic distant recurrence. The AIR of HCC was defined, as illustrated in Fig. 3, based on previous studies, as the simultaneous development of multiple nodular (at least 3) or infiltrative tumor recurrence in the treated segment of the liver, with enhancement on hepatic arterial phase images and wash-out on portal or delayed venous phase images at follow-up accompanied by a tumor thrombus in the adjacent portal vein^13^. Local recurrence without criteria for AIR was classified as non-aggressive local recurrences. At distance recurrence was defined by the appearance of a new foci of HCC in a liver segment different from the ablation zones or in the same liver sub-segment, but not adjacent to the ablation zones^16^. When both AIR and distant recurrence occurred, the patients were categorized in the AIR group.

### Histology and immunohistochemistry

In our institution since 2007, in all patients treated by percutaneous ablation for HCC a per-procedural biopsy of tumor (at least one nodule in case of multinodular form) is performed. All biopsy samples were fixed in formalin and included in paraffin and reviewed by two pathologists specialized in liver pathology (MZ, JC) to assess Edmondson-Steiner grade and histological subtype according to the WHO classification, the macro-trabecular massive (MTM) subtype, as described previously^14,17^, was reported as a distinct histological subtype (Shown in Fig. 2). Any percentage of macro-trabecular feature observed on the biopsy sample led to its classification into the MTM subtype as recommended by published criteria^14^. The MTM subtype was defined on hematein and eosin-stained sections by large trabeculae (more than 6 cells thick) lined by stromal sinusoidal cells, large trabeculae being most often discernable because separated by empty spaces. Sections (3 μm thick) from all biopsy samples were immunostained for the study with Cytokeratin 19 (CK19) and Epithelial Cell Adhesion Molecule (EpCAM) as previously described^18^. Samples were considered positive for CK19 and EpCAM when more than 5% of the tumor cells showed cytoplasmic and/or membranous staining^18^.

### Statistical analysis

Descriptive results were presented as means ± standard deviation (SD) or medians for continuous variables and as numbers (percentages) for categorical variables. Baseline characteristics were compared using Mann–Whitney test for continuous variables and χ^2^ test or Fisher’s exact test for categorical variables. Inter-observer agreement was expressed by Cohen’s kappa coefficient. Curves of time to recurrence and survival curves were built using the Kaplan–Meier method and compared using the log-rank test. The influence of baseline characteristics on AIR, local non-aggressive recurrence, at distance recurrence, overall recurrence and mortality was assessed by using Cox proportional hazards regression model in univariable analyses. Multivariable analysis was performed for all variables with a P value < 0.05 at univariable analysis. Statistical analysis was performed using the Prism7 software (Graphpad) and the R statistical software (http://www.R-project.org/).

### Informed consent and ethical approval

According to the French laws, all patients gave prospectively a written informed consent to allow the analysis of data and the analysis of tissue sample remaining after diagnosis, for a research purpose. The informed consent was approved by our Ethical Committee (Comité d'évaluation éthique de l'Inserm, IRB00003888 the 8th October 2013). Our research complies with the guidelines for human studies and was conducted ethically in accordance with the World Medical Association Declaration of Helsinki.

## Results

### Patient’s selection and baseline characteristics

One thousand seven hundred and eight patients had been referred to the multidisciplinary tumor board for HCC treatment from January 2007 to January 2017. Among them, 611 patients have been treated by no touch multi-bipolar RFA as a first line treatment. After exclusion of patients without tumoral biopsy (n = 224), of patients with a non-contributive biopsy (n = 71, 18% of all tumor biopsies) and of patients with suboptimal CT images (n = 104), 212 patients (median age, 67 years, range 34–86 years) were finally included in this study (Fig. 1).

Baseline clinical, biological, radiological and histological characteristics of the patients are described in Table 1. There were 168 men (79%) and 164 (77%) patients had a solitary tumor. Cirrhosis was histologically diagnosed in 181 patients (85.4%). In patients with multiple nodules (n = 48, 23%), the largest nodule with positive biopsy for HCC only was taken into account to assess AIR or non-aggressive local recurrence. The median tumor diameter was 2.86 cm (0.9–9) and 66 (30.4%) were larger than 30 mm. At imaging, atypical pattern of tumor vascular enhancement was observed in 27 patients (13%), non-smooth tumor margin in 45 patients (21%), tumor capsule in 109 patients (51%), a peri-tumoral enhancement at the arterial phase in 30 patients (14.3%) and a vascular contact in 86 patients (40.5%). Among the 45 patients with non-smooth tumor margin, 10 had localized (involving no more than 2 segments) infiltrative HCC. Inter-observer agreement for the assessment of non-smooth tumor margin was good (Cohen Kappa coefficient: 0.7) and excellent (Cohen Kappa coefficient: 0.79) for the assessment of capsule and abnormal peritumoral enhancement pattern. The pathological reviewing identified 66 HCC (31%) poorly differentiated with grade 3 or 4 according to Edmondson score and 25 tumors were classified as MTM (12%). Either CK19 or EpCAM were expressed in 23 HCC (11%). The median delay between imaging and treatment was 33 days.

**Table 1:** Baseline features of patients according to the type of tumor recurrence.

Variables	Available data	All patients n = 212	Aggressive intrasegmental recurrence n = 21	Local non aggressive recurrence n = 21	Distant recurrence only n = 72	Without recurrence n = 98
**Clinical features**						
Age	212	67 ± 10	69 + /- 10	70 ± 7	67 ± 9	67 ± 10
Male	212	168 (79)	18 (85.7)	15 (71)	55 (76)	76 (77)
Cirrhosis	212	181 (85.4)	17 (81)	17 (81)	68 (94)	60 (61)
Etiology of liver disease	212					
Hepatitis B		26 (12.3)	2 (10)	3 (14)	9 (12)	12 (12)
Hepatitis C		76 (35.8)	6 (28)	7 (33)	30 (42)	33 (34)
Alcohol		82 (38.7)	10 (47)	8 (38)	25 (35)	39 (40)
NASH		20 (9.4)	2 (10)	2 (10)	7 (10)	9 (9)
Other etiologies		8 (3.8)	1 (5)	1 (5)	1 (1)	5 (5)
AFP level (ng/mL)	203	108 ± 408	30 ± 60	176 ± 285	85 ± 317	127 ± 521
Child–Pugh class B	208	15 (7)	0 (0)	1(5)	4 (6)	10 (10)
Number of nodules	212					
Solitary		164 (77.3)	20 (95)	18 (85)	57 (79)	69 (70)
Multiple		48 (22.6)	1 (5)	3 (5)	15 (21)	29 (30)
Tumor size (cm)	212	2.86 ± 1.4	3.09 ± 1.32	2.98 ± 1.2	2.72 ± 1.33	2.88 ± 1.52
BCLC stage 0/A	212	199 (94)	20 (95)	21 (100)	68 (94)	90 (92)
**Imaging features**						
Atypical tumor vascular enhancement	212	27 (13)	2 (10)	1(5)	10 (14)	14 (14)
Non-smooth tumor margin	212	45 (21)	10 (47)	4 (19)	13 (18)	18 (18)
Tumor capsule	212	109 (51)	12 (57)	15 (71)	34 (47)	48 (49)
Abnormal vascular peritumoral enhancement	212	30 (14.3)	6 (28)	3 (14)	8 (11)	13 (13)
Peri-vascular location	212	86 (40.5)	11 (52)	11 (52)	24 (33)	40 (41)
**Pathological and immunohistochemical features**						
Histopathological subtype	212					
Macrotrabecular-massive		25 (12)	8 (38)	0(0)	7 (10)	10 (10)
Microtrabecular		104 (49)	6 (28)	12 (57)	43 (60)	43 (44)
Compact		5 (2.4)	0 (0)	0 (0)	2 (3)	3 (3)
Scirrhous		20 (9.4)	1 (5)	3 (14)	4 (5)	12 (12)
Steatohepatitic		42(19.8)	1(5)	5 (23)	13 (18)	23 (23)
Hepato-cholangiocarcinoma		5 (2.4)	1(5)	0 (0)	1 (1)	2 (2)
Edmondson grade 3 or 4	212	66 (31.1)	9 (43)	5 (23)	19 (26)	33 (34)
Biliary marker expression	205	23 (11.2)	1 (5)	2 (9)	7 (10)	13(14)

Results were expressed in numbers (%) or median ± standard deviation.

AFP, alphafoetoprotein level; BCLC, Barcelona Clinic Liver Classification; NASH, non-alcoholic steatohepatitis.

### Imaging and histopathological characteristics associated with different subtypes of tumor recurrence

The median follow-up was 28 months. Tumor recurrence occurred in 114 patients (54%), including AIR in 21 patients (10%), non-aggressive local recurrence in 21 patients (10%), and intra hepatic distant recurrence in 72 patients (34%). Only 2 patients had at first recurrence both AIR and at distance recurrence and those two patients were considered as having AIR for statistical analysis. Median delays for AIR, non-aggressive local recurrence and at distance recurrence were 15 months (1–44), 21 months (2–73), and 18 months (1–81), respectively. Seven patients had recurrence at first follow-up imaging. Among them, 2 patients had early AIR and 5 experienced distant recurrence. Baseline characteristics according to the occurrence of AIR, non-aggressive local tumor recurrence, or distant tumor recurrence are shown in Table 1. In the 10 patients with infiltrative tumors the incidence of AIR (3/10; 30%) was higher compared to non-infiltrative tumors (18/202; 9%), but the difference was not statistically significant p = 0.065).

AIR presented as multiple nodular tumors in 12 patients and as a diffusely infiltrative mass in 9 patients (Fig. 3). Among the 9 patients with infiltrative AIR, only one had initially an infiltrative aspect. The multiple tumor nodules were uniform in size and developed simultaneously in the same segment of the liver with contact to the ablation zone. Multiple nodular recurrences were not associated with an invasion of adjacent portal vein, whereas all tumors with an infiltrative pattern invaded the adjacent segmental portal vein. In univariable analysis, non-smooth tumor margins and MTM histological subtype were associated with a higher risk of AIR occurrence (Table 2). Multivariable analysis showed that non-smooth tumor margin at imaging (HR:3.7 [1.57–9.06]; p = 0.02) and MTM histological subtype (HR:3.8 [1.47–10]; p = 0.005) were both independently related to a higher incidence of AIR (Table 2). Univariable and multivariable analysis of overall tumor recurrence is detailed in Supplementary table 1. When considering categorical variables with tumor size > 3 cm and AFP level > 200 ng/ml, univariable and multivariable analysis of baseline characteristics associated with aggressive intra-segmental recurrence showed the same results (Supplementary table 2).

**Table 2:** Univariable and multivariable analysis of baseline characteristics associated with aggressive intra-segmental recurrence.

		Univariable analysis			Multivariable analysis		
	n	HR	95% CI	P value	HR	95% CI	P value
Age > 65 years old	212	1.79	[0.69;4.6]	0.22			
Male	212	1.55	[0.45;5.26]	0.48			
Histological diagnosis of cirrhosis	212	0.8	[0.26;2.38]	0.6			
**Etiology of liver disease**	212						
Hepatitis B		0.83	[0.11;5.95]	0.8			
Hepatitis C		0.76	[0.15;3.79]	0.7			
Alcohol		1.25	[0.27;5.74]	0.7			
Other		1.31	[0.11;14.48]	0.8			
AFP level (ng/mL)	205	0.99	[0.99;1.00]	0.3			
Child–Pugh class B	211	0.96	[0.1;9.28]	0.9			
Solitary nodule	212	5.12	[0.68;38.19]	0.11			
Tumor size (cm)	212	1.01	[0.98;1.04]	0.35			
BCLC stage B	212	0.96	[0.1;9.28]	0.9			
Atypical pattern of tumor enhancement	212	1.34	[0.3;5.7]	0.69			
Non-smooth tumor margin	212	4.8	[2.03;11.31]	0.0003	3.7	[1.57;9.06]	0.002
Tumor capsule	212	1.17	[0.49;2.79]	0.7			
Abnormal vascular peritumoral enhancement	212	2.5	[0.99;6.61]	0.051			
Irregular circumferential enhancement		2.54	[0.74;8.63]	0.13			
Peri-vascular location	212	1.66	[0.7; 3.91]	0.24			
MTM subtype	212	6.14	[2.53;14.88]	0.00005	3.8	[1.47;10]	0.005
Edmondson grade 1 or 2		0.52	[0.22;1.24]	0.44			
Biliary marker expression	201	0.57	[0.07;4.27]	0.58			

AFP, alphafoetoprotein level; BCLC, Barcelona Clinic Liver Classification; HR, hazard ratio; MTM, macrotrabecular massive.

### Survival analysis

**Figure 4:**
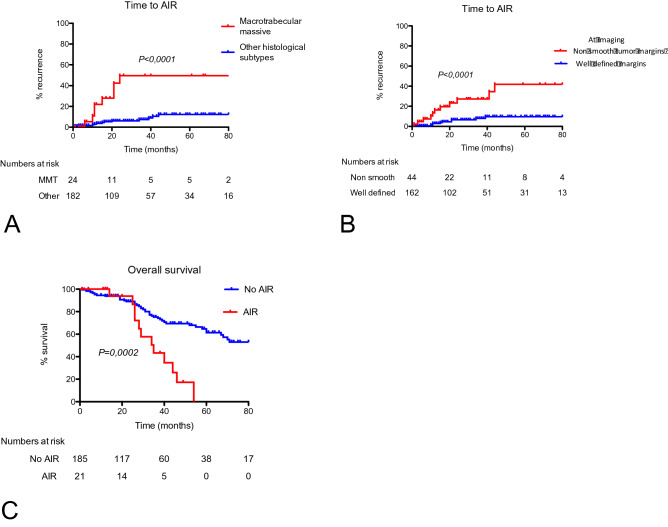
Time to aggressive intrasegmental recurrence according to radiological and histological features and overall survival according to the presence of aggressive intrasegmental recurrence. Kaplan–Meier curves for AIR according to the macrotrabecular massive subtype at histology (**A**), and non smooth tumor margin at imaging (**B**). Kaplan–Meier curves for survival according to the occurrence of an aggressive intrasegmental recurrence (**C**). Statistical analysis was performed using the log rank test. The numbers at risk were reported under the x axis. The cox-proportional hazard assumption is fulfilled in the analyses.

In the whole series, 70 (33%) patients died and 4 were transplanted at the end of follow-up. The median overall survival was 70 months. Prognosis was strongly influenced by AIR with a median overall survival of 35 months in patients with AIR versus 85 months in patients without AIR (log-rank test, p = 0.0001, Fig. 4). Histological diagnosis of cirrhosis, Child–Pugh class B, BCLC stage B, non-smooth tumor margin at imaging and MTM histological subtype, were related to poor survival in univariable analysis (Table 3). In multivariable analyses, histological cirrhosis (HR:3.35 [1.15–9.37]; p:0.02), Child–Pugh class B (HR:2.6 [1.09–6.16]; p:0.03), BCLC stage B (HR:2.6 [1.09–6.16]; p:0.03), and MTM histological subtype (HR:2.12 [1.04–4.32]; p:0.03) remained independently related to a higher risk of death (Table 3).

**Table 3:** Univariable and multivariable analysis of baseline characteristics associated with overall survival.

		Univariable analysis			Multivariable analysis		
	n	HR	95% CI	P value	HR	95% CI	P value
Age > 65 years old	212	1.04	[0.83;2.39]	0.2			
Male	212	0.9	[0.51;1.83]	0.9			
Histological cirrhosis	212	2.92	[1.06;8.07]	0.03	3.35	[1.15;9.37]	0.02
**Etiology of liver disease**	212	Ref					
Hepatitis B		1.13	[0.4;3.2]	0.8			
Hepatitis C		0.74	[0.29;1.86]	0.5			
Alcohol		1.19	[0.49;2.88]	0.7			
Other		0.33	[0.04;2.79]	0.31			
AFP level (ng/mL)	205	1	[0.99;1.00]	0.5			
Child–Pugh class B	208	2.71	[1.23;5.99]	0.01	2.54	[1.06;6.04]	0.03
Solitary nodule	212	0.58	[0.33;1.03]	0.06			
Tumor size (cm)	212	0.99	[098;1.01]	0.97			
BCLC stage B	212	2.95	[1.3;6.54]	0.007	2.6	[1.09;6.16]	0.03
Atypical pattern of tumor enhancement	212	1.04	[0.47;2.30]	0.91			
Non-smooth tumor margin	212	1.77	[1.02;3.07]	0.04	1.58	[0.86;2.88]	0.13
Tumor capsule	212	0.7	[0.43;1.19]	0.2			
Abnormal vascular peritumoral enhancement	212	0.49	[0.18;1.3]	0.17			
Irregular circumferential enhancement		0.25	[0.03;1.8]	0.17			
Peri-vascular location	212	0.98	[0.58;1.6]	0.95			
MTM subtype	212	2.07	[1.05;4.1]	0.03	2.12	[1.04;4.32]	0.03
Edmondson grade 1 or 2		0.83	[0.48;1.44]	0.5			
Biliary marker expression	201	1.78	[0.84;3.76]	0.13			

AFP, alphafoetoprotein level; BCLC, Barcelona Clinic Liver Classification; HR, hazard ratio; MTM, macrotrabecular massive.

### Correlation between imaging and histological features

Given the prognosis value of MTM histological subtype, we analyzed associated radiological features. The presence of a peritumoral vascular enhancement was significantly associated with the MTM subtype (p = 0.003, Table 4).

**Table 4:** Baseline clinical and imaging characteristics associated with macrotrabecular-massive subtype.

Variables	Available data (n)	All patients n = 212	Patients with MTM n = 25	Patients without MTM n = 187	p
Age > 65 years old	212	125 (59)	16 (64)	109 (58)	0.68
Male (%)	212	168 (79)	19 (76)	149 (80)	0.46
Histological cirrhosis (%)	212	181 (85)	18 (72)	163 (87)	0.06
Etiology of liver disease (%)	212				0.7
Hepatitis B		26 (12.3)	2 (9)	2 (8.3)	
Hepatitis C		76 (35.8)	6 (28)	5 (20.8)	
Alcohol		82 (38.7)	11 (52)	10 (41.6)	
NASH		20 (9.4)	1 (4.7)	2 (8.3)	
Other		8 (3.8)	1 (4.7)	5 (20.8)	
AFP level (ng/mL)	203	64.3	99 ± 29	168 ± 103	0.42
Child–Pugh class B (%)	208	15 (7)	2 (8)	13 (6)	0.7
Solitary	212	164 (77)	22 (88)	142 (76)	0.21
Tumor size (cm)	212	2.8 ± 1	3.2 ± 2,5	2,8 ± 1	0.18
BCLC stage B	208	15 (7)	2 (5)	13 (7)	0.7
Atypical pattern of tumor vascular enhancement	212	28 (13)	3 (9.5)	2(9)	0.64
Non-smooth tumor margin	212	46 (21.7)	8 (32)	38 (20)	0.19
Tumor capsule	212	109 (51)	16 (64)	93(49)	0.2
Peri-vascular location	212	86 (40)	12 (48)	74 (65)	0.5
Peritumoral vascular enhancement	212	30 (16)	9 (36)	21 (11)	0.003
Edmondson grade 1 or 2	212	146 (69)	10 (40)	136 (72)	0.002
Biliary marker expression	201	23 (11)	4 (17)	19 (10)	0.3

Statistical analysis were performed using the Mann–Whitney test and the Fisher exact test.

AFP, alphafoetoprotein level; BCLC, Barcelona Clinic Liver Classification; HR, hazard ratio; MTM, macrotrabecular massive subtype at histology; NASH, non-alcoholic steatohepatitis.

### Subgroup analysis restricted to patients with small nodular tumors

**Figure 5:**
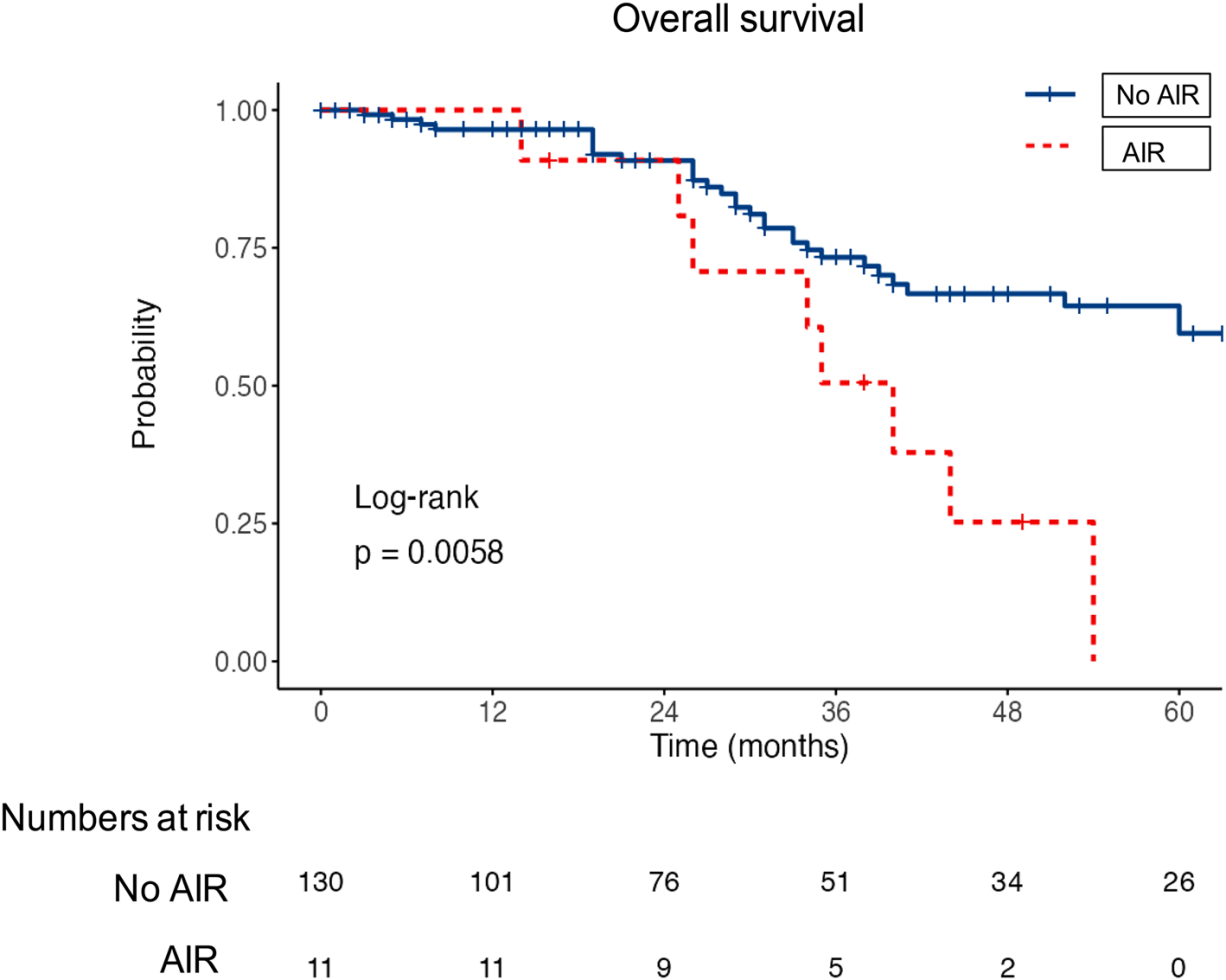
Overall survival according to the presence of aggressive intrasegmental recurrence in the subgroup of patients with nodules smaller than 3 cm and without infiltrative tumors. The cox-proportional hazard assumption is fulfilled in the analyses.

A subgroup analysis was performed, excluding patients with infiltrative localized HCC and patients with tumor larger than 3 cm (n = 142). In this subgroup, 11 AIR occurred (8%). Patients with AIR had a worst outcome survival compared to those without AIR (Log-rank p = 0.0058; Fig. 5). Pre-treatment factors independently associated with overall survival were the CHILD Pugh B status (HR:4.42 [1.53;12.78]; p = 0.006) and MTM subtype at histology (HR:3.60 [1.63;7.97]; p = 0.002) (Table 5). If the variable AIR was added to the multivariable analysis as a prognostic factor, the CHILD Pugh B status and the presence of MTM subtype at histology remained independently associated to survival while AIR was associated with survival only in the univariable analysis (Supplementary Table 4). MTM remained associated to AIR in this subgroup of patients (p = 0.014).

**Table 5:** Univariable and multivariable analysis of characteristics associated with overall survival in patient with non-infiltrative HCC smaller than 3 cm.

		Univariable analysis			Multivariable analysis		
	n	HR	95% CI	P value	HR	95% CI	P value
Age > 65 years old	142	1.57	[0.83;2.96]	0.165			
Male	142	0.96	[0.44;2.07]	0.909			
Histological cirrhosis	142	2.75	[0.66–11.45]	0.164			
**Etiology of liver disease**	142						
NASH	16	ref					
Hepatitis B	14	1.27	[0.34;4.74]	0.724			
Hepatitis C	58	1.21	[0.40;3.70]	0.735			
Alcohol	49	2.06	[0.70;6.09]	0.191			
Other	5	0.00	NA	0.996			
AFP level (ng/mL)	142	1	[1.00;1.00]	0.445			
Child–Pugh class B	131	3.65	[1.28;10.36]	0.015	4.42	[1.53;12.78]	0.006
Solitary nodule	142	0.80	[0.39;1.62]	0.530			
Tumor size (cm)	142	1.01	[0.95;1.06]	0.849			
BCLC stage A	142	1.09	[0.52;2.28]	0.821			
Atypical pattern of tumor enhancement	142	0.56	[0.13;2.32]	0.423			
Non-smooth tumor margin	142	1.46	[0.70;3.07]	0.313			
Tumor capsule	142	0.59	[0.32;1.11]	0.103			
Abnormal vascular peritumoral enhancement	142	0.42	[0.10;1.74]	0.231			
Irregular circumferential enhancement	142	0.59	[0.32;1.11]	0.103			
Peri-vascular location	142	1.01	[0.52;1.96]	0.970			
MTM subtype	142	3.19	[1.46;6.95]	0.004	3.60	[1.63;7.97]	0.002
Edmondson grade 1 or 2	142	0.77	[0.40;1.49]	0.441			
Biliary marker expression	139	1.61	[0.57;4.54]	0.365			

NASH, non-alcoholic steatohepatitis; AFP, alphafoetoprotein level; BCLC, Barcelona Clinic Liver Classification; HR, hazard ratio; MTM, macrotrabecular massive.

The incidence of AIR in patients with HCC smaller than 3 cm was 12/146 (8.2%) compared to 9/66 (13.6%) in patients with HCC greater than 3 cm, the difference was not statistically relevant (p = 0.2). Similarly, the incidence of AIR in patients with HCC smaller than 5 cm was 19/199 (10%) compared to 2/13 (15.3%) in patients with HCC greater than 5 cm, the difference was not statistically relevant (p = 0.6).

## Discussion

Based on the analysis of radio-pathological features of HCC in 212 patients treated by multipolar radiofrequency ablation, we identified that two baseline radiological and histological features represented by non-smooth borders and MTM subtype were predictive of AIR, an aggressive tumor recurrence pattern.

Local recurrence eligible for re-ablation is prognostically completely different from AIR. AIR has been rarely described under this name^13,19^ but is related to the critical notion of time to interventional failure (elapsed time from resection to unresectable/unablatable recurrence) that dramatically affect survival^20^. We demonstrated in this study that AIR was strongly associated with a high risk of death in this population of patients, and suggests that a better understanding of the determinants of AIR would be helpful to improve clinical care and prognosis after RFA. Compared to the 2 studies previously reporting AIR after ablation procedures^13,19^ the frequency observed in our study was higher (10% versus 3.7% and 3.2% respectively). These discrepancies may be related to the fact that we took into account recurrences occurring before 6 months post RFA (3 out of 21 in our study) because we considered that such early and aggressive tumor progression occurring in spite of the use of highly effective ablative method as multi-bipolar RFA, relies mostly on the primary aggressiveness of the tumor, regardless of the delay of its detection. Moreover, there are some major differences in the baseline features and treatment, since both studies^13,19^ included patients 10 years younger, mostly affected by HBV virus, mostly without tumor biopsy, and with different ablation technique (monopolar RFA or microwave ablation), that do not allow a direct comparison on AIR incidence. In our study, perivascular localization, either portal or venous, was not associated with AIR, neither in univariable, nor in multivariable analysis. This could be related to the use of multi-bipolar radiofrequency ablation that was able to avoid the “heat sink effect” and lead to the complete ablation in HCC in the vicinity of major vessels, as demonstrated in previous studies^9,21,22^. On the other hand, we found, as described in both studies^13,19^ that the size of the tumor was not associated with AIR. Thus, the least influence of tumor size and proximity of large vessels using multi-bipolar radiofrequency compared to monopolar RFA may have limited the impact of these factors on AIR, which in our series seems to be rather imputable to the aggressive histological characteristics of the tumor.

Non-smooth border margin at imaging was also independently associated with AIR in our study. The presence of non-smooth border margin has been repeatedly identified as an independent predictor of HCC recurrence after surgical resection^23,24^ and tightly related to microvascular invasion. We show here that this feature indicates a worst prognosis also in multi-bipolar RFA. We also showed that the MTM subtype was a strong predictor of AIR. Previous studies have showed that MTM, and a very similar pattern frequently observed within MTM HCC called “Vessels that encapsulate tumor clusters” (VETC)^16^, were associated with high risk of tumor recurrence and death in patients treated by liver resection and RFA, but none of these previous studies have linked histology with this aggressive pattern of recurrence^14,25,26^. The aggressive local behavior of MTM as well as its link with microvascular invasion suggest that this histological subtype has angioinvasion properties without features of epithelia-mesenchymal transition^14,27^. Moreover, MTM has been shown to bear a strong prognostic value in surgically resected HCC patients, independently of microvascular invasion^13,16,22^. In contrast to microvascular invasion, MTM is easily assessed on tumor biopsy with an excellent interobserver agreement according to previous studies.

Patients with localized infiltrative HCC, and with tumor larger than 3 cm, that were considered as eligible for a curative treatment by multipolar RFA by our multidisciplinary tumor board, have been included in this study, but would have a different prognosis. Therefore, considering the subgroup of patients with non-infiltrative tumors smaller than 3 cm, we confirmed that MTM histological subtype remained the unique tumor marker independently associated to AIR and to a reduced overall survival. As expected, when considering tumors below 30 mm without infiltrative borders, the radiological feature “non-smooth tumor margins” was no more associated with the overall survival nor with AIR. This is supporting our hypothesis that AIR remains an event essentially influenced by the underlying tumor biology even in the case of small tumors with well-defined borders. Our data support the usefulness of pretreatment tumor biopsy. Interestingly, we observed an enrichment of abnormal vascular peritumoral enhancement at imaging in MTM HCC. This is in agreement with the findings of Rhee et al.^28,29^, who reported that irregular rim-like arterial phase hyperenhancement was tightly associated with histological macrotrabecular pattern. Interestingly the same researchers confirmed a similar pattern of enhancement at MRI in a Korean multicenter cohort of MTM-HCC; the detection of an arterial phase internal or diffuse hypovascular component in association with tumor peripheral or hypervascular foci reported a significant association with MTM-HCC^28,29^. According with these findings another French monocentric study reported that substantial necrosis could independently predict MTM-HCCs^30^. In our series, no necrosis was observed and this could be explained by the smaller size of HCC treated by RFA compared to surgically treated HCC of other studies^30^.

Even if our study has some limitations, notably the retrospective design and the presence of a systematic per procedural tumor biopsy not usually performed worldwide, considering the poorer outcome associated with the presence of MTM-HCC, we believe that tumor biopsy could be discussed during the diagnosis work-up.

In our study the biopsy did not lengthen the time from diagnosis to treatment (below 5 weeks), previously described as a time associated with a negative outcome^31^. The presence of pejorative tumor features such as non-smooth border margin at imaging or MTM histological subtypes could be used to propose alternative treatments such as anatomic resection if feasible, larger ablation area in using multi-bipolar segmental ablation technique, or neoadjuvant or adjuvant systemic therapy.

## Supplementary Information

Supplementary Table 1.

Supplementary Table 2.

Supplementary Table 3.

Supplementary Table 4.

## Data Availability

All data generated or analyzed during this study are included in this article [and/or] its supplementary material files. Further enquiries can be directed to the corresponding author (Pr. Marianne Ziol). The scientific guarantor of this publication is Pr. Marianne Ziol (Last author).

## Abbreviations

RFA: Radiofrequency ablation
mbpRFA: Multi-bipolar RFA
HCC: Hepatocellular carcinoma
BCLC: Barcelona Clinic Liver Cancer
MTM: Macrotrabecular massive
AFP: Alpha-foeto-protein
US: Ultrasonography
CT: Computerized tomography
MR: Magnetic resonance
EpCAM: Epithelial cell adhesion molecule
AIR: Aggressive intrasegmental recurrence
NASH: Non-alcoholic steatohepatitis
HR: Hazard ratio
