# Supplementary material for: Imaging and histological features of tumor biopsy sample predict aggressive intrasegmental recurrence of hepatocellular carcinoma after radiofrequency ablation
Source: Sci Rep. 2022 Nov 4;12:18712. doi: 10.1038/s41598-022-23315-5 (PMC9636258; doi:10.1038/s41598-022-23315-5)
Supplement: Supplementary file 1 — Supplementary Table 1. [file 41598_2022_23315_MOESM1_ESM.docx]

Supplementary table 1: Univariable and multivariable analysis of baseline characteristics associated with overall recurrence

|  |  | Univariable analysis | | | Multivariable analysis | | |
| --- | --- | --- | --- | --- | --- | --- | --- |
|  | n | HR | 95% CI | P value | HR | 95% CI | P value |
| Age ­> 65 years old | 212 | 1.42 | [0.99;1.03] | 0.07 |  |  |  |
| Male | 212 | 0.91 | [0.57;1.46] | 0.7 |  |  |  |
| Histological diagnosis of cirrhosis | 212 | 2.08 | [1.14;3.8] | 0.01 | 2.69 | [1.39;5.22] | 0.003 |
| Etiology of liver disease | 212 |  |  |  |  |  |  |
| Hepatitis B |  | 0.91 | [0.40;2.04] | 0.8 |  |  |  |
| Hepatitis C |  | 0.95 | [0.48;1.85] | 0.8 |  |  |  |
| Alcohol |  | 1.18 | [0.43;1.63] | 0.6 |  |  |  |
| Other |  | 2.19 | [0.12;1.65] | 0.2 |  |  |  |
| AFP level (ng/mL) | 205 | 0.99 | [0.99;1.00] | 0.4 |  |  |  |
| Child-Pugh class B | 211 | 1.80 | [0.78;4.14] | 0.16 |  |  |  |
| Solitary nodule | 212 | 1.28 | [0.77;2.13] | 0.32 |  |  |  |
| Tumor size >30 mm | 212 | 1.06 | [0.70;1.58] | 0.7 |  |  |  |
| BCLC stage B | 212 | 1.15 | [0.45;2.96] | 0.7 |  |  |  |
| Atypical pattern of tumor enhancement | 212 | 1.02 | [0.56;1.86] | 0.94 |  |  |  |
| Non-smooth tumor margin | 212 | 1.59 | [1.03;2.4] | 0.03 | 1.23 | [0.77;1.98] | 0.37 |
| Tumor capsule | 212 | 1.002 | [0.68;1.45] | 0.99 |  |  |  |
| Abnormal vascular peritumoral enhancement | 212 | 1.07 | [0.63;1.79] | 0.78 |  |  |  |
| Peri-vascular location | 212 | 1.08 | [0.74;1.58] | 0.6 |  |  |  |
| MTM subtype | 212 | 2.01 | [1.16;3.5] | 0.01 | 1.6 | [0.87;2.96] | 0.12 |
| Edmondson grade 1 or 2 |  | 0.89 | [0.59;1.33] | 0.5 |  |  |  |
| Biliary marker expression | 201 | 1.03 | [0.54;1.99] | 0.9 |  |  |  |

AFP = alphafoetoprotein level, BCLC = Barcelona Clinic Liver Classification, HR = hazard ratio, MTM= macrotrabecular massive
