# Supplementary material for: Imaging and histological features of tumor biopsy sample predict aggressive intrasegmental recurrence of hepatocellular carcinoma after radiofrequency ablation
Source: Sci Rep. 2022 Nov 4;12:18712. doi: 10.1038/s41598-022-23315-5 (PMC9636258; doi:10.1038/s41598-022-23315-5)
Supplement: Supplementary file 2 — Supplementary Table 2. [file 41598_2022_23315_MOESM2_ESM.docx]

Supplementary table 2: Univariable and multivariable analysis of baseline characteristics associated with aggressive intra-segmental recurrence considering tumor size > 3 cm and AFP level > 200 ng/ml

|  |  | Univariable analysis | | | Multivariable analysis | | |
| --- | --- | --- | --- | --- | --- | --- | --- |
|  | n | HR | 95% CI | P value | HR | 95% CI | P value |
| Age ­> 65 years old | 212 | 1.79 | [0.69;4.6] | 0.22 |  |  |  |
| Male | 212 | 1.55 | [0.45;5.26] | 0.48 |  |  |  |
| Histological diagnosis of cirrhosis | 212 | 0.8 | [0.26;2.38] | 0.6 |  |  |  |
| Etiology of liver disease | 212 |  |  |  |  |  |  |
| Hepatitis B |  | 0.83 | [0.11;5.95] | 0.8 |  |  |  |
| Hepatitis C |  | 0.76 | [0.15;3.79] | 0.7 |  |  |  |
| Alcohol |  | 1.25 | [0.27;5.74] | 0.7 |  |  |  |
| Other |  | 1.31 | [0.11;14.48] | 0.8 |  |  |  |
| AFP level > 200 ng/ml | 205 | 2.4 | [0.32;17.6] | 0.4 |  |  |  |
| Child-Pugh class B | 211 | 0.96 | [0.1;9.28] | 0.9 |  |  |  |
| Solitary nodule | 212 | 5.12 | [0.68;38.19] | 0.11 |  |  |  |
| Tumor size > 3 cm | 212 | 2.01 | [0.85- ;4.78] | 0.114 |  |  |  |
| BCLC stage B | 212 | 0.96 | [0.1;9.28] | 0.9 |  |  |  |
| Atypical pattern of tumor enhancement | 212 | 1.34 | [0.3;5.7] | 0.69 |  |  |  |
| Non-smooth tumor margin | 212 | 4.8 | [2.03;11.31] | 0.0003 | 3.7 | [1.57;9.06] | 0.002 |
| Tumor capsule | 212 | 1.17 | [0.49;2.79] | 0.7 |  |  |  |
| Abnormal vascular peritumoral enhancement | 212 | 2.5 | [0.99;6.61] | 0.051 |  |  |  |
| Irregular circumferential enhancement |  | 2.54 | [0.74;8.63] | 0.13 |  |  |  |
| Peri-vascular location | 212 | 1.66 | [0.7 ; 3.91] | 0.24 |  |  |  |
| MTM subtype | 212 | 6.14 | [2.53;14.88] | 0.00005 | 3.8 | [1.47;10] | 0.005 |
| Edmondson grade 1 or 2 |  | 0.52 | [0.22;1.24] | 0.44 |  |  |  |
| Biliary marker expression | 201 | 0.57 | [0.07;4.27] | 0.58 |  |  |  |

AFP = alphafoetoprotein level, BCLC = Barcelona Clinic Liver Classification, HR = hazard ratio, MTM= macrotrabecular massive.
