# Supplementary material for: Imaging and histological features of tumor biopsy sample predict aggressive intrasegmental recurrence of hepatocellular carcinoma after radiofrequency ablation
Source: Sci Rep. 2022 Nov 4;12:18712. doi: 10.1038/s41598-022-23315-5 (PMC9636258; doi:10.1038/s41598-022-23315-5)
Supplement: Supplementary file 3 — Supplementary Table 3. [file 41598_2022_23315_MOESM3_ESM.docx]

Supplementary Table 3: Univariable and multivariable analysis of baseline characteristics associated with overall survival with the addition of AIR

|  |  | Univariable analysis | | | Multivariable analysis | | |
| --- | --- | --- | --- | --- | --- | --- | --- |
|  | n | HR | 95% CI | P value | HR | 95% CI | P value |
| Age ­>65 years old | 212 | 1.04 | [0.83;2.39] | 0.2 |  |  |  |
| Male | 212 | 0.9 | [0.51;1.83] | 0.9 |  |  |  |
| Histological cirrhosis | 212 | 2.92 | [1.06;8.07] | 0.03 | 3.36 | [1.17;9.63] | 0.024 |
| Etiology of liver disease | 212 | Ref |  |  |  |  |  |
| Hepatitis B |  | 1.13 | [0.4;3.2] | 0.8 |  |  |  |
| Hepatitis C |  | 0.74 | [0.29;1.86] | 0.5 |  |  |  |
| Alcohol |  | 1.19 | [0.49;2.88] | 0.7 |  |  |  |
| Other |  | 0.33 | [0.04;2.79] | 0.31 |  |  |  |
| AFP level (ng/mL) | 205 | 1 | [0.99;1.00] | 0.5 |  |  |  |
| Child-Pugh class B | 208 | 2.71 | [1.23;5.99] | 0.01 | 2.67 | [1.11;6.44] | 0.028 |
| Solitary nodule | 212 | 0.58 | [0.33;1.03] | 0.06 |  |  |  |
| Tumor size (cm) | 212 | 0.99 | [098;1.01] | 0.97 |  |  |  |
| BCLC stage B | 212 | 2.95 | [1.3;6.54] | 0.007 | 2.82 | [1.17;6.79] | 0.021 |
| Atypical pattern of tumor enhancement | 212 | 1.04 | [0.47;2.30] | 0.91 |  |  |  |
| Non-smooth tumor margin | 212 | 1.77 | [1.02;3.07] | 0.04 | 1.37 | [0.74;2.52] | 0.316 |
| Tumor capsule | 212 | 0.7 | [0.43;1.19] | 0.2 |  |  |  |
| Abnormal vascular peritumoral enhancement | 212 | 0.49 | [0.18;1.3] | 0.17 |  |  |  |
| Irregular circumferential enhancement |  | 0.25 | [0.03;1.8] | 0.17 |  |  |  |
| Peri-vascular location | 212 | 0.98 | [0.58;1.6] | 0.95 |  |  |  |
| MTM subtype | 212 | 2.07 | [1.05;4.1] | 0.03 | 1.73 | [0.83;3.61] | 0.143 |
| Edmondson grade 1 or 2 |  | 0.83 | [0.48;1.44] | 0.5 |  |  |  |
| Biliary marker expression | 201 | 1.78 | [0.84;3.76] | 0.13 |  |  |  |
| AIR | 211 | 3.10 | [1.62-5.95] | 0.001 | 2.68 | [1.25;5.75] | 0.012 |
