# Supplementary material for: Imaging and histological features of tumor biopsy sample predict aggressive intrasegmental recurrence of hepatocellular carcinoma after radiofrequency ablation
Source: Sci Rep. 2022 Nov 4;12:18712. doi: 10.1038/s41598-022-23315-5 (PMC9636258; doi:10.1038/s41598-022-23315-5)
Supplement: Supplementary file 4 — Supplementary Table 4. [file 41598_2022_23315_MOESM4_ESM.docx]

Supplementary Table 4: Univariable and multivariable analysis of characteristics associated with overall survival in patient with non-infiltrative HCC smaller than 3 cm with the addition of AIR

|  |  | Univariable analysis | | | Multivariable analysis | | |
| --- | --- | --- | --- | --- | --- | --- | --- |
|  | n | HR | 95% CI | P value | HR | 95% CI | P value |
| Age ­>65 years old | 142 | 1.57 | [0.83;2.96] | 0.165 |  |  |  |
| Male | 142 | 0.96 | [0.44;2.07] | 0.909 |  |  |  |
| Histological cirrhosis | 142 |  | [0.83;2.96] |  |  |  |  |
| Etiology of liver disease | 142 |  |  |  |  |  |  |
| NASH | 16 | ref |  |  |  |  |  |
| Hepatitis B | 14 | 1.27 | [0.34;4.74] | 0.724 |  |  |  |
| Hepatitis C | 58 | 1.21 | [0.40;3.70] | 0.735 |  |  |  |
| Alcohol | 49 | 2.06 | [0.70;6.09] | 0.191 |  |  |  |
| Other | 5 | 0.00 | NA | 0.996 |  |  |  |
| AFP level (ng/mL) | 142 | 1.00-1.00 | [] | 0.445 |  |  |  |
| Child-Pugh class B | 131 | 3.65 | [1.28;10.36] | 0.015 | 4.65 | [1.60;13.51] | 0.005 |
| Solitary nodule | 142 | 0.80 | [0.39;1.62] | 0.530 |  |  |  |
| Tumor size (cm) | 142 | 1.01 | [0.95;1.06] | 0.849 |  |  |  |
| BCLC stage A | 142 | 1.09 | [0.52;2.28] | 0.821 |  |  |  |
| Atypical pattern of tumor enhancement | 142 | 0.56 | [0.13;2.32] | 0.423 |  |  |  |
| Non-smooth tumor margin | 142 | 1.46 | [0.70;3.07] | 0.313 |  |  |  |
| Tumor capsule | 142 | 0.59 | [0.32;1.11] | 0.103 |  |  |  |
| Abnormal vascular peritumoral enhancement | 142 | 0.42 | [0.10;1.74] | 0.231 |  |  |  |
| Irregular circumferential enhancement | 142 | 0.59 | [0.32;1.11] | 0.103 |  |  |  |
| Peri-vascular location | 142 | 1.01 | [0.52;1.96] | 0.970 |  |  |  |
| MTM subtype | 142 | 3.19 | [1.46;6.95] | 0.004 | 2.67 | [1.10;6.47] | 0.030 |
| Edmondson grade 1 or 2 | 142 | 0.77 | [0.40 ;1.49] | 0.441 |  |  |  |
| Biliary marker expression | 139 | 1.61 | [0.57;4.54] | 0.365 |  |  |  |
| AIR | 141 | 2.89 | [1.31-6.37] | 0.008 | 2.24 | [0.87;5.73] | 0.093 |
